# Circulating tumour cell RNA characterisation from colorectal cancer patient blood after inertial microfluidic enrichment

**DOI:** 10.1016/j.mex.2019.06.012

**Published:** 2019-06-17

**Authors:** Marnie Winter, Zhen Cai, Katharina Winkler, Kristen Georgiou, Daniel Inglis, Tina Lavranos, Meysam Rezaei, Majid Warkiani, Benjamin Thierry

**Affiliations:** a Future Industries Institute and ARC Centre of Excellence in Convergent Bio-Nano Science and Technology, University of South Australia, Mawson Lakes Campus, Mawson Lakes, South Australia, Australia; b Laboratory Medicine Center, NanFang Hospital, Southern Medical University, Guangzhou, China; c Bionomics, Thebarton, South Australia, Australia; d School of Biomedical Engineering, University of Technology Sydney, Sydney, Ultimo, NSW, Australia; e Institute of Molecular Medicine, Sechenov First Moscow State University, Moscow, Russia

**Keywords:** Circulating tumour cells isolation and analysis with droplet digital PCR, Circulating tumour cell, Droplet digital PCR, Colorectal cancer

## Abstract

The detection and molecular analysis of circulating tumour cells (CTCs) potentially provides a significant insight to the characterisation of disease, stage of progression and therapeutic options for cancer patients. Following on from the protocol by Warkiani et al. 2016, which describes a method of enriching CTCs from cancer patient blood with inertial microfluidics, we describe a method to measure the CTC RNA expression in the enriched fraction using droplet digital PCR and compare transcript detection with and without RNA pre-amplification.

• Inertial microfluidics combined with droplet digital PCR is advantageous as it allows for CTC enrichment and subsequent RNA analysis from patient blood. This allows for patient tumour analysis with increased sensitivity and precision compared to quantitative Real Time PCR and enables the direct quantification of nucleic acids without the need for tumour biopsy.
• This method demonstrates an efficient approach providing important insights into the analysis of colorectal cancer patients’ CTCs using a specific gene subset or biomarkers, an approach that may be tailored to tumour type or expanded to larger panels.

## Graphical abstract

**Figure:**
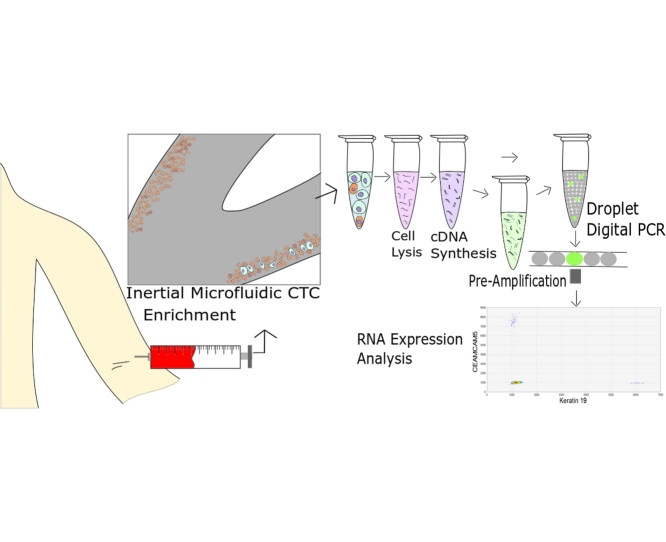

**Specifications Table**

**Table:** 

Subject Area:	*Biochemistry, Genetics and Molecular Biology*
More specific subject area:	*Digital Molecular Biology*
Method name:	*Circulating tumour cells isolation and analysis with droplet digital PCR*
Name and reference of original method:	Warkiani, Majid Ebrahimi, Bee Luan Khoo, Lidan Wu, Andy Kah Ping Tay, Ali Asgar S. Bhagat, Jongyoon Han, and Chwee Teck Lim. "Ultra-fast, label-free isolation of circulating tumor cells from blood using spiral microfluidics." *Nature protocols*11, no. 1 (2016): 134.
Resource availability:	*n/a*

## Method details

### Background

It has been well established that circulating tumour cells (CTCs) isolated from the blood of cancer patients provide the potential for non-invasive prognostic and molecular insights [[1], [2], [3]]. Historically CTC enumeration has been used as an independent prognostic indicator, however, enumeration alone does not provide molecular information or therapeutic options for the patients’ disease. The enrichment and detection of smaller numbers of CTCs leading to earlier identification of disease and detection of relapse has allowed the approach to shift towards molecular characterisation of CTCs to better guide patient treatment, provide better prognostics and monitor response to therapy [4]. There has been significant research in developing technologies to enrich CTCs. These approaches often include immuno-isolation which rely solely on the expression of epithelial markers. However, the metastatic portion of cells are often in a state of epithelial to mesenchymal transition [5] meaning that prognostically relevant CTCs may not express epithelial markers (in fact, mesenchymal cells are often associated with poorer patient prognosis [6,7]). Therefore, an immuno-unbiased enrichment approach based on the CTCs’ physical features may be preferable towards objective characterization of the disease molecular phenotype. Inertial microfluidics provides an immuno-unbiased CTC enrichment strategy which has previously been shown to yield an 80% recovery of cancer cell line spiked in to healthy blood. Inertial microfluidics has been applied successfully in the enrichment of CTCs in a number of cancer types including advanced stage metastatic breast and lung [8,9]. As well as enabling the processing of relatively large volumes of blood after red blood cell (RBC) lysis (>7.5 mL) in a few minutes, inertial microfluidics also allows for downstream analyses using various techniques including immunofluorescence, cell culture, fluorescence *in situ* hybridisation (FISH), quantitative real time – polymerase chain reaction (qRT-PCR) and even single cell analysis as previously outlined [10,11]. As described by Warkiani and colleagues, inertial microfluidic devices work via inherent Dean vortex flows present in curvilinear microchannels under continuous flow, and through inertial lift forces. This results in smaller particles being trapped in the Dean vortices towards the outer channel walls which are removed by the outer outlet, while the larger particles (CTCs) equilibrate near the inner channel wall and are collected from the inner outlet [9].

Droplet digital PCR (ddPCR) is advantageous as it has been shown to have increased sensitivity and precision over traditional qRT-PCR, providing direct quantification of nucleic acids from a wider range of samples [12]. Importantly, as it does not require standard curves, it provides an absolute measure of nucleic acid content with greatly increased sensitivity of detection [13]. DdPCR has been used for the molecular detection and analyses of CTCs in a number of studies in combination with several enrichment techniques (e.g. magnetic beads) [[14], [15], [16]]. Therefore, the combination of immuno-unbiased size based high throughput CTC enrichment provided by inertial microfluidics and the sensitivity of ddPCR is ideal for the molecular characterization of CTCs isolated from patients’ blood.

Following on from the published protocol by Warkiani et al. which describes a method of enriching CTCs from patient blood with inertial microfluidics [17], we describe a method to measure the expression of CTC RNA in the enriched fraction using ddPCR with and without RNA pre-amplification. Initially, we used cancer cell lines spiked into healthy blood to validate and optimise ddPCR for the specific panel of gene markers indicative of epithelial or tumour cells. After optimisation, the technique of inertial microfluidic enrichment and ddPCR for the detection of the specific markers of interest was performed on a small number of colorectal cancer patient samples. The presence of CTC specific markers was confirmed in the samples, demonstrating the utility of this protocol.

### CTC isolation

CTCs were isolated using a trapazoidal spiral inertial microfluidic device as previously described [17]. The trapazoidal spiral inertial microfluidic device design and fabrication has previously been described [9,17]. Moulds for chips were fabricated using standard micromilling at the South Australian or New South Wales nodes of the Australian National Fabrication Facility with each being subjected to a strict quality control protocol.

a) 9 ml blood samples were collected (for both healthy persons and CRC patient blood) in a cell free DNA BCT tube (218962; Streck, Nebraska, USA), stored at room temperature and processed within 24 h.
b) Red blood cells were lysed using a commercial lysis buffer (gBioscience RBC Lysis buffer; 786–849) with 35 ml of lysis buffer used per 9 ml of blood. Samples were placed on a gentle shaker for 15 min at room temperature before centrifugation for 10 min at 500 g with a slow brake.
c) Supernatant was removed from the cell pellet and the pellet resuspended in PBS (14190-144, Gibco™) (all pipette tips were pre-coated with 0.2% Poly(ethylene glycol)-block-poly(propylene glycol)-block-poly)ethylene glycol)(Pluronic®F-108, Sigma-Aldrich; 542342). The pellet was resuspended in 3× volume of PBS than initial blood volume (i.e. 9 ml blood in 27 ml PBS).
d) Microfluidic device enrichment was performed as described in Warkiani et al. [17]. Briefly, the device channels were coated with 1% bovine serum albumin and the resuspended pellet was injected (TYD01-02, Leadfluid) through the slanted inertial microfluidic device at a constant rate of 1700 μl/min.
e) The enriched fraction was collected and pelleted with centrifugation for 10 min at 500 g.
f) The supernatant was removed, the cell pellet resuspended in in 500 μl of RNA later and stored at −80 °C until analysis.

Note: Streck cell free DNA BCT tubes were used for this purpose as they contain a light preservative which allows for storage and shipping of samples without significant cellular degradation [18].

### RNA extraction

a) The enriched sample (in RNA later) was centrifuged for 10 min at 3,500 *g*, and the RNAlater removed.
b) 100 μl of extraction buffer was added to the pellet as per manufacturer’s instructions (Picopure™ RNA Isolation kit, Thermofisher, KIT0204) and was mixed well.
c) The sample and extraction buffer was incubated for 30 min at 42 °C.
d) The Picopure column was activated by adding 250 μl of conditioning buffer for 5 min at room temperature. The column was then centrifuged at for 1 min 16,200 *g*.
e) 100 μl of ethanol (from the Picopure kit) was added to the 100 μl of extraction buffer (RNA mixture), the sample was mixed well, added to column and centrifuged for 2 min at 100 *g*.
f) 100 μl of washing buffer #1 from Picopure kit was added to column and centrifuged for 1 min at 8000 *g*.
g) 100 μl of washing buffer #2 from Picopure kit was added to column and centrifuged for 1 min at 8000 *g*. Next, 100 μl of washing buffer #2 was again added to column and centrifuged for 2 min at 16,000 *g* and then 1 min at 16,000 *g*.
h) 11.5 μl of elution buffer was then added to column, incubated for 1 min and centrifuged for 1 min at 16,000 *g*.
i) 1.5 μl of the 10x ezDNAse buffer, 1 μl of nuclease free water and 1.5 μl of the ezDNAse were added to 11 μl of RNA as per the manufacturer’s instructions (11766051, Thermofisher). Sample was incubated for 2 min at 37 °C and then placed at 4 °C.
j) RNA quantity was measured using the Qubit RNA HS Assay Kit as per the manufacturer’s instructions (Q32852, Thermofisher). New standards were prepared before each set of readings (10 μl of standard and 190 μl of buffer). 3 μl of ezDNAse treated RNA sample was added to 1 μl of dye and 196 μl of buffer and the concentration was read using a Qubit 2.0 fluorometer (Thermofisher).

Note: The presence of genomic contamination was monitored when analysing extracted RNA samples. For our set-up it was found with the Picropure RNA isolation kit alone that occasionally there was significant genomic contamination. Therefore, various methods (ezDNAse, Turbo DNA-free™, Purelink RNA mini kit, Thermofisher) for additional DNAse treatment were investigated. DNAse treatment time was also investigated and determined that 2 min was adequate to prevent genomic contamination. ezDNAse (Thermofisher) was selected as the best method to eliminate the issue of genomic contamination.

### cDNA synthesis

a) As per manufacturer’s instructions, per sample 4 μl of 5x iScript advanced reaction mix, 1 μl of iScript reverse transcriptase (1725038, BioRad) and 4 μl of nuclease free water was added to 11 μl of ezDNAse treated RNA.
b) Sample was placed in thermocycler and was run as per the following protocol: 20 min 46 °C, 1 min 95 °C and held at 4 °C.

### Pre-amplification

Due to the low amount of CTCs in enriched samples, we investigated the use of pre-amplification to increase the detectable amount of target RNAs. Pre-amplification was performed with Taqman pre-amplification master-mix as per manufacturer’s instructions (Thermofisher Scientific). For each blood sample pre-amplification was performed on 10 μl of cDNA. In this instance, we amplified using probes from the selected panel of CRC markers: KRT-19, CEACAM5, AGR2, FDZ7 and LGR5.

Briefly for each sample, 25 μl of 20× Taqman preamp master mix (4391128, Thermofisher) was added to 12.5 μl of probe mix (1 μl of each probe LGR5, CEACAM5, KRT19 (Keratin 19), AGR2, FDZ7 in 95 μl Tris-EDTA (TE) buffer (AM9849, Thermofisher)) and then 2 μl of nuclease free water and 10 μl of cDNA was added. The sample was then placed in the thermocycler and run as per the following protocol (10 min 95 °C then 15 s 95°c and 4 min 60 °C repeated 9× and held at 4 °C). Pre-amplified solution was again diluted with dH_2_O 1:5 for running with the ddPCR.

**Fig. 1:**
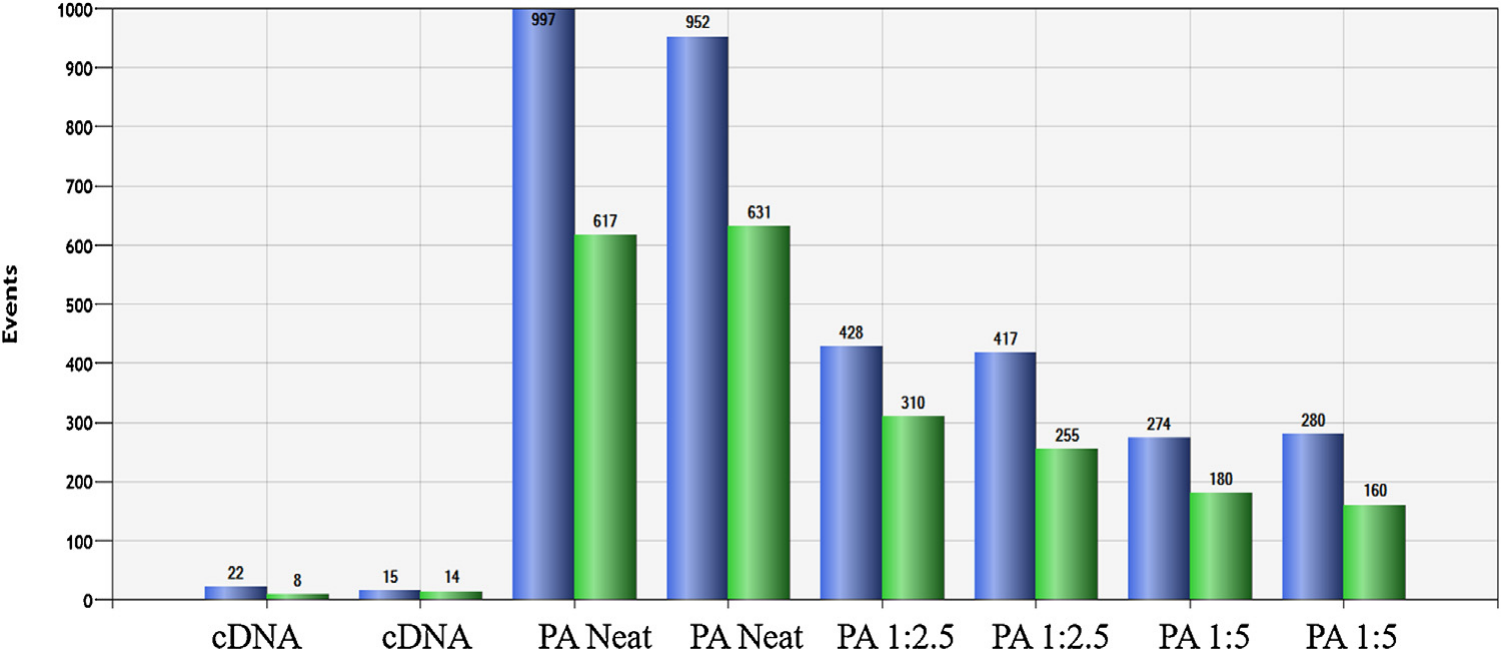
Column graph representing total positive droplet numbers for KRT19 (blue) and and CEACAM5 (green) for 10 LoVo cells in 100,000 WBC for samples without pre-amplification (designated cDNA) and those with pre-amplification (PA) and indicated dilutions. For all subsequent experiments pre-amplified samples were diluted 1:5 as per the manufacturer’s instructions, as well as providing a larger volume for analysis dilution may potentially prevent precipitation which could in turn cause channel blockage during droplet generation and reduce the number of droplets.

To investigate pre-amplification utility we firstly used 10 or 50 of the ATCC® CCL-229™ LoVo cells (colon, derived from left supraclavicular region metastasis) either in the presence or absence of WBCs to simulate normal enriched conditions. To quantitate efficiency, we used the Qubit 2.0 fluorometer for measurement of high sensitivity (HS) RNA (Q32852) and single stranded DNA (ssDNA; Q10212). However, the ssDNA measurement is only relevant if the sample does not have background WBC contamination (as the pre-amplification will only amplify targets spiked into the mix). Therefore, we determined the best way to measure efficiency was to use ddPCR. We directly compared the same sample before and after amplification and it was found that 20–100× more positive droplets were obtained after amplification (Fig. 1). Subsequently, patients’ samples were analyzed with and without pre-amplification, in order to obtain a quantitative measurement (without pre-amplification) and a qualitative measurement (with pre-amplification) with multiple markers and a greater number of replicates (which is afforded by preamplification and is perhaps the biggest advantage).

### Droplet digital PCR

For each probe, the optimum annealing/extension temperature was investigated with a temperature gradient. It was found that 1 min for 60 °C was optimum for all probes in the expected temperature range for the assay. The optimum concentration for each probe was also investigated and it was found that 1.1 μl of each probe per reaction gave maximal results.

Initially, probes were run individually with cDNA from either 10 or 10,000 ATCC® HT-29 (HTB-38™colorectal adenocarcinoma derived, epithelial cells) cells (or LoVo cells) and expression was quantified. Two probes were then used together per analysis sample enabling the simultaneous detection on the FAM and HEX channels, either KRT19 and CEACAM5 or LGR5 and AGR2/FDZ7. The copies/μl of the target was compared alone and in combination. It was determined that the use of two specific probes in combination did not affect copy numbers/μl and could be used for analysis.

1 For each well, 1.1 μl of probe (2 probes per well, CEACAM5 FAM and KRT19 HEX or AGR2 FAM, LGR5 HEX), 11 μl of ddPCR supermix (1863024, Bio Rad), 4 μl nuclease free water, 4.8 μl cDNA for a final volume of 22 μl was added and mixed well. The plate was sealed with Biorad Aluminium plate seal (#1814040) and the plate was centrifuged for 30 s at room temperature.
2 The plate was placed in the QX200™ AutoDG™ droplet generation robot (Bio Rad) and set up as per manufacturer’s instructions.
3 After droplet generation, the plate was placed into thermocycler (Eppendorf deep well thermocycler) as per instructions for ddPCR Probe assay (PrimePCR™ ddPCR™ Gene Expression Probe Assays; Bio Rad). i.e. enzyme activation 95 °C for 10 min, denaturation for 15 s 94 °C, annealing/extension for 1 min 60 °C (denaturation/extension repeated 39 cycles), enzyme deactivation 98 °C and hold at 4 °C with a ramp rate of 2 °C/sec.
4 The plate was then placed in QX200 Droplet digital PCR system and run as an absolute quantification experiment as per manufacturer’s instructions.
5 Analysis was then performed in QuantaLife (Bio Rad).

Note: FAM is considered a brighter fluorophore than HEX and therefore, probes were used to match expected expression levels i.e. high expressing KRT19 was run with HEX while CEACAM5 was run with FAM.

Note: Multiplexing with more than 2 probes involves mixing different amounts of FAM and HEX probes in various proportions to enable detection. The use of 5 probe multiplexing was investigated, however, proved problematic and unreliable for this specific application (due to the extreme rarity of markers) and it was ultimately decided to limit to 2 probes per reaction.

Note: It is understood that in ddPCR false positive droplets will occur in no-template control runs and for negative samples (blood samples prepared in the same way as patient samples but are from a healthy control, constitutes ˜100,000 WBC). It is therefore, important to determine false positive rates for each probe and no template controls are continually run to determine the presence of reagent contamination and other errors. False positive rates assume all sources, droplets, detection, assays and contamination. False positive rate is calculated as number of events per well and used to calculate the limit of detection. False positive rate is different for each probe and must be calculated independently. We initially investigated FDZ7 as a marker of CTCs, however, it was found that the expression of this marker was too high in WBCs to detect CTC expression, as has been previously described [19]. Therefore, it was decided that FDZ7 would not be used for further analysis.

Note: We observed that pre-amplification significantly increases the number of false positive events (except for CEACAM5 which remained low) and therefore, while there is increase in the number of positive droplets, there is also a higher threshold over which a well can be considered truly positive (Table 1). Therefore, the number of false positives was investigated with and without pre-amplification. For both cDNA and pre-amplified cDNA false positivity rate was calculated. To calculate this negative control wells were run for each probe and the total number of positive droplets was enumerated.

$$False\;positve\;rate=\frac{Total\;number\;of\;positive\;droplets}{Number\;of\;wells}$$

**Table 1:** False positive rates (FPR) for the four markers investigated (CEAMCAM5, KRT19, LGR5 and AGR2) for both pre-amplified and unamplified cDNA.

	CEACAM5		KRT19		LGR5		AGR2	
	FPR	Positive Droplets	FPR	Positive Droplets	FPR	Positive Droplets	FPR	Positive Droplets
Pre-amplified	0.595	3	7.420	22	7.615	22	32.075	96
cDNA	0.045	2	0.200	3	–	–	–	–

Based on the false positive rate, the number of positive droplets required per well to be a considered a true positive (with 95% confidence was calculated based on data table available from Bio-Rad (California, USA). For CEACAM5 which has a false positive rate of 0.6%, the threshold to assess a positive well was 3 positive droplets for 95% certainty that the result is a true positive. For KRT19 which has a 7.4% false positive rate, the threshold to assess a positive well is 22 positive droplets for 95% certainty that the result is a true positive. For LGR5 which has a 7.4% false positive rate the threshold to assess a positive well is 22 positive droplets for 95% certainty that the result is a true positive. For AGR2 which has a 32.1% false positive rate, the threshold to assess a positive well is 96 droplets for 95% certainty. This number of droplets is unrealistic and it is likely that these samples do not represent a true negative, therefore, similar to FDZ7, AGR2 is not a feasible marker to aid in the detection of CRC CTCs in this instance, although its utility has however, previously been demonstrated as a marker for prostate cancer CTCs [20].

### Method validation

#### Detection sensitivity

**Fig. 2:**
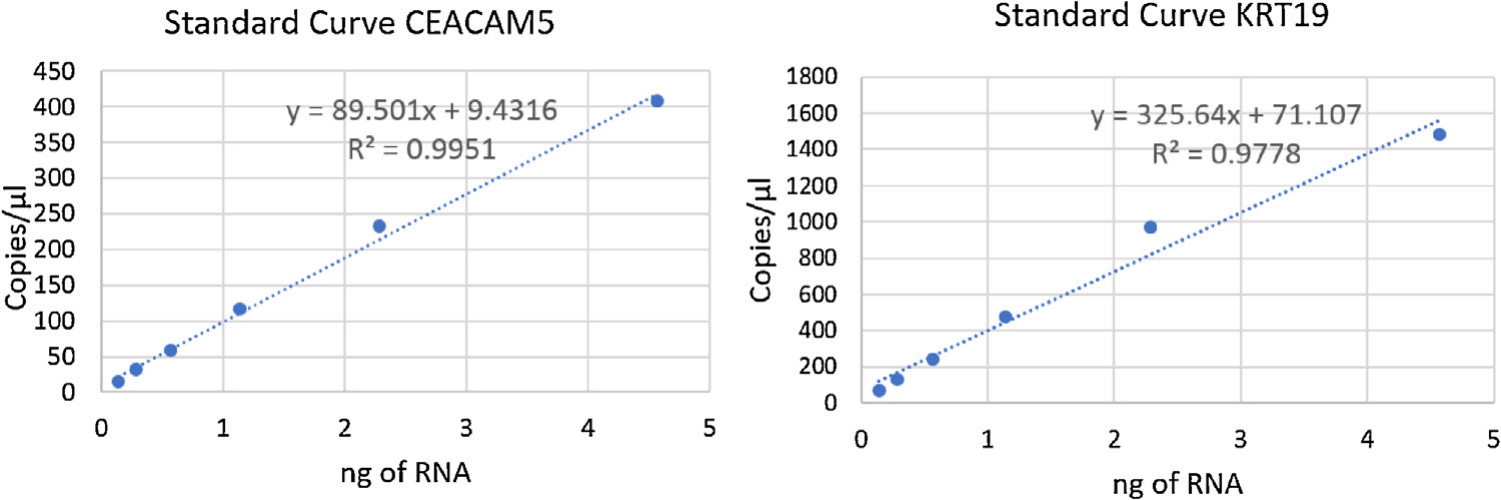
Standard curves to clarify the relationship between number of cells (amount of RNA) and the copy number per μL for both CEACAM5 and KRT19 calculated based on cDNA dilutions from LoVo cDNA.

Using a dilution series, as few as 12 LoVo cells could be detected in 1 well using the CEACAM5 probe (Fig. 2). As low as 3 LoVo cells per well with the KRT19 probe could be detected attributed to high KRT19 expression levels in this cell type (Table 2). However, it is noteworthy that the minimum number of CTCs detectable in a patient sample will be higher as KRT19 expression is expected to be lower than in cancer cell lines.

**Table 2:** Number of positive droplets detected with various LoVo cell numbers in presence of WBC background (100,000) with or without pre-amplification (PA).

	KRT19	LGR5	CEACAM5
20 LoVo cells in WBC	0 ± 0	0 ± 0	0 ± 0
20 LoVo cells in WBC (PA)	25.57 ± 5.10	33.25 ± 6.50	0 ± 0
50 LoVo in WBC	21 ± 4.55	33 ± 5.00	8.33 ± 3.68
50 LoVo in WBC (PA)	274.25 ± 3.90	298.50 ± 2.50	165.75 ± 7.26

#### Spiking experiments and patient samples

Experiments spiking both HT-29 and LoVo cells into equivalent numbers of WBC background that is typically obtained following inertial microfluidic enrichment were first performed. It was found that the detection sensitivity was reduced with the presence of WBCs. Moreover, the presence of WBCs considerably increased the number of false positives (hence increasing the number of positive droplets needed to be considered a true positive) and decreased the limit of detection. Low numbers of LoVo cells (20 cells) could be detected within a background WBC population from 9 ml of enriched blood.

Blood samples from CRC patients were processed as per the described protocol and expression of CTC markers was measured (Supplementary Information Table 1). The presence of CTC specific markers (hence CTCs) was confirmed in some of the patient samples in 1 or multiple samples from the same patient. This study has shown that mRNA expression of genes specific to epithelial or tumor cells can be detected in colorectal cancer patient samples.

### Summary

This article has described a method to assess mRNA expression of genes specific to epithelial or tumour cells with ddPCR after inertial microfluidic enrichment of CTCs from patient blood samples. It is shown that this method can be used to detect colorectal cancer patient CTCs. The detection of potential CTCs with ddPCR is made possible through the initial size based enrichment with the inertial microfluidic device as originally described by Warkiani et al. [17].
