# Supplementary material for: Circulating tumour cell RNA characterisation from colorectal cancer patient blood after inertial microfluidic enrichment
Source: MethodsX. 2019 Jun 17;6:1512–20. doi: 10.1016/j.mex.2019.06.012 (PMC6603295; doi:10.1016/j.mex.2019.06.012)
Supplement: Supplementary file 1 [file mmc1.docx]

**Circulating tumour cell RNA characterisation from colorectal cancer patient blood after inertial microfluidic enrichment.**

Marnie Winter, Zhen Cai, Katharina Winkler, Kristen Georgiou, Daniel Inglis, Tina Lavranos, Meysam Rezaei, Majid Warkiani, Benjamin Thierry

**Supplementary material:**

**RNA Extraction**

Note: Initially the SingleShot™ Cell Lysis Kit (Bio Rad, 1725080) was selected to eliminate the risk of RNA loss with a column based system. For RNA extraction after removal of RNAlater, 50 µl of lysis buffer was added to enriched cell fraction and incubated for 10 minutes at room temperature, followed by 5 minutes at 37^o^C and 5 minutes at 75^o^C. cDNA synthesis was then performed using the iScript cDNA synthesis kit from BioRad as previously outlined. For each sample 20 µl of cDNA was synthesised but a maximum of 5 µl of cDNA could be used for ddPCR per well as the droplet generation is particularly sensitive to the components of the system. It was found that for this purpose, the use of SingleShot lysis buffer (we hypothesise due to salts in the buffers) resulted in inadequate numbers of droplet generation and therefore, was not suitable for this purpose. We therefore, decided to use a column based extraction system to minimise the presence of salts that can interfere with droplet generation.

Supplementary Table 1: Results from colorectal cancer patient enriched blood samples including Qubit measurements and copies/µl, number of positive droplets for keratin19 (KRT), CEACAM5, LGR5 and AGR2 for both samples that did not undergo preamplification (cDNA) or pre-amplified (PA). For each patient up to 4 replicate blood samples were performed (a, b, c and d).
